# Does Alcohol Cue Inhibitory Control Training Survive a Context Shift?

**DOI:** 10.1037/adb0000580

**Published:** 2020-04-13

**Authors:** Andrew Jones, Laura Baines, Helen Ruddock, Ingmar Franken, Frederick Verbruggen, Matt Field

**Affiliations:** 1 Department of Psychological Sciences, University of Liverpool; 2 School of Psychology, University of Birmingham; 3 Department of Psychology, Education and Child Studies, Erasmus University; 4 Department of Experimental Psychology, Ghent University; 5 Department of Psychology, University of Sheffield

**Keywords:** alcohol, inhibitory control training, stimulus value, stop-signal task

## Abstract

Inhibitory control training (ICT) is a novel psychological intervention that aims to improve inhibitory control in response to alcohol-related cues through associative learning. Laboratory studies have demonstrated reductions in alcohol consumption following ICT compared with control/sham training, but it is unclear if these effects are robust to a change of context. In a preregistered study, we examined whether the effects of ICT would survive a context shift from a neutral context to a seminaturalistic bar setting. In a mixed design, 60 heavy drinkers (40 female) were randomly allocated to receive either ICT or control/sham training in a neutral laboratory over 2 sessions. We developed a novel variation of ICT that used multiple stop signals to establish direct stimulus–stop associations. The effects of ICT/control were measured once in the same context and once following a shift to a novel (alcohol-related) context. Our dependent variables were ad libitum alcohol consumption following training, change in inhibitory control processes, and change in alcohol value. ICT did not reduce alcohol consumption in either context compared with the control group. Furthermore, we demonstrated no effects of ICT on inhibitory control processes or alcohol value. Bayesian analyses demonstrated overall support for the null hypotheses. This study failed to find any effects of ICT on alcohol consumption or candidate psychological mechanisms. These findings illustrate the difficulty in training alcohol-inhibition associations and add to a growing body of literature suggesting that ICT holds little evidential value as a psychological intervention for alcohol use disorders.

Alcohol use disorders are associated with impairments in the ability to suppress inappropriate behavior (Smith, Mattick, Jamadar, & Iredale, 2014; Yücel et al., 2019), commonly known as inhibitory control (Logan, Cowan, & Davis, 1984). This failure to inhibit behavior can be measured using stop-signal or go/no-go tasks (Eagle, Bari, & Robbins, 2008; Verbruggen & Logan, 2008a). In these tasks, participants are required to make speeded motor responses to cues that appear on a majority of trials. On a minority of trials, the presence of a “stop signal” or a “go/no-go” cue requires inhibition of the motor response. Poor inhibitory control can be inferred using commission errors (failure to inhibit) and stop-signal reaction time (the unobserved latency of inhibition; Verbruggen et al., 2019; Verbruggen & Logan, 2008b).

Among people who consume alcohol, impairments in reactive inhibitory control are reliably exacerbated during exposure to alcohol-related cues (Jones, Robinson, et al., 2018). This transient impairment is thought to arise because alcohol-related cues have appetitive motivational properties, and they evoke approach behaviors that are incompatible with inhibition (Field, Kiernan, Eastwood, & Child, 2008; Field, Mogg, & Bradley, 2005). The failure of inhibitory control in response to alcohol-related cues may increase the likelihood of drinking behavior because inhibition is required to overcome the approach behaviors triggered by these cues (de Wit, 2009; Jones, Christiansen, Nederkoorn, Houben, & Field, 2013). Consistent with this view, transient inhibitory impairments may mediate ad libitum alcohol consumption after exposure to alcohol cues (Field & Jones, 2017). Furthermore, in alcohol-dependent patients, the magnitude of inhibitory impairment in response to alcohol cues predicts the likelihood of relapse following treatment (Czapla et al., 2016).

Although reactive inhibitory control has provided the basis for the majority of research into alcohol use, flexible human control is also proactive in nature, requiring careful planning and strategic adjustments (Elchlepp, Lavric, Chambers, & Verbruggen, 2016). For example, when people are attempting to reduce their alcohol consumption, it is implausible to inhibit all motor movement (a global stopping response). Rather, they should adopt a proactive strategy in anticipation of being exposed to alcohol-related cues (Aron, 2011). Following a failure (or inefficient use) of proactive control, reactive stopping may be employed as a last resort or late-correction mechanism during self-regulation (Braver, Paxton, Locke, & Barch, 2009). *Strategic* proactive control adjustments can be inferred by the degree of slowing of reaction times in contexts in which an inhibitory signal is anticipated (see Verbruggen & Logan, 2009; Verbruggen, Stevens, & Chambers, 2014), suggesting that proactive control is a top-down process and is influenced by the expectation of future inhibition (see Baines, Field, Christiansen, & Jones, 2019; Best, McLaren, & Verbruggen, 2019). There have been some attempts to examine the role of proactive control in alcohol use disorders, but this warrants further investigation (see Baines et al., 2019).

The observation that alcohol-related cues impair reactive control and that these impairments increase the likelihood of alcohol consumption has led to the development of a novel behavioral intervention, known as inhibitory control training (ICT), that is designed to improve inhibitory control in response to alcohol-related cues. During ICT, participants complete a modified stop-signal or go/no-go task that includes alcohol-related and neutral cues. In the active training group, participants are trained to respond quickly to neutral cues, whereas an inhibitory signal (stop signal or no-go cue) is paired with the majority of (or all) alcohol-related cues. Therefore, through associative learning, participants should learn that alcohol-related cues require an inhibitory response and become more efficient at inhibiting behavior in the presence of these cues (see Jones & Field, 2013). Control groups are either not required to make inhibitory responses or they are exposed to reversed response contingencies such that they are instructed to make motor responses to alcohol-related cues while inhibiting to neutral/control images. Following training, participants’ motivation to drink alcohol is examined using ad libitum consumption paradigms (see Jones, Button, et al., 2016) in which free access to alcohol is provided. Numerous studies have demonstrated that active ICT, compared with control groups, prompts reduced alcohol consumption in the laboratory (Bowley et al., 2013; Di Lemma & Field, 2017; Houben, Havermans, Nederkoorn, & Jansen, 2012; K. Houben, Nederkoorn, Wiers, & Jansen, 2011; Jones & Field, 2013), with a small- to medium-size effect (Allom, Mullan, & Hagger, 2016; Jones, Di Lemma, et al., 2016).

Although improvements in alcohol-related inhibitory control are the proposed mechanism of action of ICT, empirical support for this claim is mixed. Jones and Field (2013) demonstrated improvements in inhibitory control to alcohol cues following ICT but did not examine whether this mediated the effect on alcohol consumption. Furthermore, Houben et al. (2012) demonstrated that ICT using a modified go/no-go task did not improve inhibitory control to alcohol-related cues on a stop-signal task. A second, nonmutually exclusive proposed mechanism of ICT is the devaluation of alcohol-related cues through repeated inhibitory responses to those cues. Behavior stimulus interaction theory, as proposed by Veling, Holland, and van Knippenberg (2008), suggests that through repeatedly inhibiting to stimuli that normally evoke approach tendencies (alcohol cues; see previous discussion), a response conflict emerges. To resolve this conflict, negative affect is attached to the stimuli, meaning that they are evaluated less positively, thereby facilitating inhibition. In support of this mechanism, Houben et al. (2012) demonstrated that ICT reduced the positive evaluation of alcohol-related cues, and this mediated the reduction in alcohol consumption following training. However, there have been failures to replicate this finding (Bowley et al., 2013; Di Lemma & Field, 2017).

Some important issues have reduced enthusiasm for ICT as a technique for the reduction of alcohol consumption and other motivated behavior (Jones, Hardman, Lawrence, & Field, 2018). First, there are emerging null effects in the published literature, which suggests that estimates of the average effect size in meta-analyses have been overestimated because of publication bias and small sample sizes (Adams, Lawrence, Verbruggen, & Chambers, 2017; Smith, Dash, Johnstone, Houben, & Field, 2017). Second, effect sizes could have been inflated by comparison to control conditions that encourage responding to alcohol cues while inhibiting to neutral cues. These conditions should strengthen associations between alcohol cues and approach and thereby increase the subjective value of those cues (Schonberg et al., 2014). Finally, any effects of ICT on drinking behavior are seemingly short-lived and easily abolished once participants leave the laboratory (Allom et al., 2016; Bowley et al., 2013; Jones & Field, 2013).

One possible reason for these short-lived effects relates to the associative-learning principles that are thought to underlie the effects of ICT on behavior. Learned associations (e.g., alcohol → inhibition) are thought to be context dependent (Bouton, 2004; Rosas, Todd, & Bouton, 2013). This is particularly evident for extinction learning, which does not erase original learned responses but, rather, suppresses them in the extinction context. Original responses may be renewed in a new context (known as AAB renewal, where B is the renewal of behavior in a new context after it is changed in the original context [A]; see Bouton, 2014). Any attempt to translate ICT into a viable behavioral intervention requires ICT to be administered across numerous environmental contexts. The rationale for this is that each context will contain different stimulus elements, and increasing the breadth of those elements during ICT will increase the likelihood that any novel context will contain at least some elements that are associated with extinguished responding, thereby reducing the likelihood of the renewal of appetitive alcohol associations.

A recent randomized controlled trial (Jones, McGrath, et al., 2018) examined whether multiple sessions of Internet-delivered ICT combined with a brief intervention (Down Your Drink; Linke, Brown, & Wallace, 2004) led to reductions in alcohol consumption over a 4-week period in heavy drinkers who were motivated to reduce their drinking. This study demonstrated substantial nonspecific reductions in alcohol consumption but no beneficial effect of ICT compared with a control intervention. The study also demonstrated little support for any proposed mechanism of ICT, including improvements in general or cue-specific inhibitory control or devaluation of alcohol-related stimuli. These disappointing results could have arisen because the ICT training procedure involved only a single stop signal (a red *=* in this study), which may have been suboptimal for the training of stimulus–stop associations (as described later in the article). Furthermore, the study did not establish whether participants completed the training sessions in contexts in which they typically consumed alcohol (e.g., in the living room at home or in pubs or bars) or in contexts in which alcohol was not typically consumed (e.g., in the bedroom or office).

Therefore, if ICT is to yield beneficial effects on alcohol intake, attempts must be made to increase the robustness of training effects such that they can survive a shift in context. According to some associative-learning theories of ICT, there are two potential pathways by which ICT works: a direct and an indirect pathway. The direct pathway suggests that an alcohol cue can directly signal an inhibition response (alcohol → inhibition), whereas the indirect pathway suggests an alcohol cue primes the detection of a stop signal, which increases the likelihood of successful inhibition if a stop signal is detected (alcohol → signal → inhibition; see Bowditch, Verbruggen, & McLaren, 2016; Verbruggen, Best, Bowditch, Stevens, & McLaren, 2014). This distinction is important: If ICT influences alcohol consumption via the latter indirect pathway, there are unlikely to be any beneficial effects of ICT on alcohol consumption when alcohol consumers are in contexts that are devoid of stop signals (i.e., all contexts in which alcohol is consumed outside of the laboratory). It is relevant that all of the existing alcohol (and food) ICT studies used single inhibition signals, which may favor the development of indirect (cue → signal → inhibition) associations that are less likely to persist outside of the training context. However, it is possible to train direct (cue → inhibition) associations by using multiple different stop signals during training (Best, Lawrence, Logan, McLaren, & Verbruggen, 2016; Bowditch et al., 2016).

The primary aim of the present study was to apply associative-learning theory to increase the likelihood that the effects of alcohol ICT would persist following a shift from a neutral training context to a novel (alcohol-related) context. We designed an ICT paradigm in which the signal or rule to inhibit changed over a series of blocks (based on Best et al., 2016). In our control group, participants were required to respond to alcohol cues on 50% of trials but inhibit responding on the remaining 50%. We applied these 50:50 contingencies to reduce the likelihood of inadvertently training alcohol-approach associations and thereby overcome weaknesses in previous ICT studies. We examined whether the anticipated effects of ICT in the training context would persist following a shift to the novel alcohol-related context during a subsequent testing session. In addition, we attempted to isolate the effects of ICT on proactive control as a potential mechanism of action, which has yet to be investigated. The presence of proactive-control adjustments for stop-associated alcohol cues would indicate that ICT effects are more strategic than initially thought (Best et al., 2019). We hypothesized that ICT, compared with the control group, would (a) reduce alcohol consumption when administered in both the same context and following a shift in context, (b) lead to an increase in reactive stopping and proactive slowing to alcohol-related cues when tested in both the same context and following a shift in context, and (c) lead to devaluation of alcohol-related cues when tested in both the same context and following a shift in context.

## Method

The study was preregistered, and data are available on the Open Science Framework (https://osf.io/snp8d). The experiment employed a 2 (Group: ICT vs. Control; between-subjects) × 2 (Context: Training Context vs. Novel Context; within-subjects) design. Across two sessions, participants completed a stop-signal task that measured proactive control and reactive control before and after ICT or control training. In each session, participants completed a measure of ad libitum alcohol consumption after ICT/control training. To minimize demand characteristics, the experiment used a cover story (“Taste Perception and Cognitive Performance in Different Contexts”).

### Participants

Sixty participants (40 female) were recruited, with a mean age of 25.33 ± 6.82 years (range: 18–45 years). The study was powered was to detect a medium effect size (*f* = .25) for a mixed analysis of variance (ANOVA; within–between interaction), with 90% power, α = .05 and 10% missing data. Participants were recruited from the university and local community via advertisements placed on the Internet and local media. Eligibility criteria required individuals to be aged 18+, to drink in excess of UK government guidelines (14 units of alcohol per week; a guide to UK alcohol units was provided in the online advertisements) on a regular basis and self-report liking beer. Participants were excluded if they self-reported a history of substance use disorder and/or other psychiatric disorders. Participants had to be sober at the time of testing, confirmed in all participants by a blood alcohol content (BAC) reading of 0 at the beginning of each session. The study was approved by the local research ethics committee.

### Materials

#### Stop-signal task

Each trial began with the presentation of a fixation cross (*+*) for 500 ms in the center of the screen. This was immediately followed by an alcohol-related image, presented in portrait or landscape orientation. Images were taken from our previous studies (Di Lemma & Field, 2017), and each depicted alcoholic beverages or models drinking alcohol. Participants had to identify the orientation of the image by pressing a key (V = portrait, N = landscape) on the keyboard as quickly as possible (“go trials”). In the majority of trials, this was uninterrupted. However, in “stop trials,” two horizontal red lines (*=*; the stop signal) were superimposed over the image, and participants were instructed to inhibit their motor response when this happened. The stop-signal delay was set at 250 ms at the beginning of each block and followed a dynamic staircase procedure in which the delay increased by 50 ms for every successful inhibition (maximum: 1,150 ms) and decreased by 50 ms for every failed inhibition (minimum: 0 ms).

The stop-signal task consisted of three blocks: no-signal block, low-probability block, and high-probability block. In the no-signal block, participants completed 40 go trials only (no stop trials). In the low-probability block, participants completed 90 go trials and 30 stop trials (75%/25% probability, respectively). In the high-probability block, participants completed 60 go trials and 60 stop trials (50%/50% probability, respectively). Blocks occurred in a random order across sessions and participants, as did trial types within blocks. All participants completed a short practice block of 10 trials. The task took approximately 15 min to complete.

#### Inhibitory control training/control (based on Houben et al., 2011)

We used a go/no-go task for ICT because this yields the largest effects on alcohol consumption in laboratory studies (Jones, Di Lemma, et al., 2016). Participants were shown alcohol and neutral images in the center of the screen (the same images from the stop-signal task were used), and target stimuli were superimposed on top of the images. There were four blocks of the task in which the response rule changed on each block. In one block, participants had to respond to lowercase letters (*h* and *r*) and inhibit to uppercase letters (*H* and *R*); in a second block, participants had to respond to consonants (*t* and *n*) and inhibit to vowels (*a* and *e*); in a third block, participants had to respond to two different symbols (*£@* and *@£*) and inhibit to symbols that were the same (*££* and *@@*); and in a fourth block, participants had to respond to numbers higher than 5 (*6* and *8*) and inhibit to numbers lower than 5 (*2* and *4*). Blocks were counterbalanced across participants and sessions.

There were 200 trials in each block. Participants in the ICT group had to inhibit on 90% of trials (90 trials) during the presentation of an alcohol cue (responding on 10% or 10 trials) and inhibit on 10% of trials (10 trials) during the presentation of a neutral cue (responding on 90% or 90 trials). In the control group, participants were required to inhibit on 50% of trials (50 trials) during the presentation of alcohol cues and 50% of trials (50 trials) with neutral cues; for the remaining trials, they had to respond. In between each block, participants were asked to complete a word search for a variable amount of time (between 1 and 5 min), in an attempt to reduce the spontaneous recovery of previous alcohol approach/inhibition associations by spacing out training blocks (see Bouton, 2002). Across all training blocks, there were 360 alcohol-inhibition pairings in the ICT group. The training task took approximately 40 min to complete.

#### Stimulus-value task (based on Chen, Veling, Dijksterhuis, & Holland, 2016)

Participants were shown the 20 alcohol-related images from the training task in the center of the screen and asked to rate how attractive each image was (“How attractive do you do you find this image?”) using a visual analogue scale with the anchors *Not at all* and *Extremely* presented underneath. The midpoint of the line was at 0, with *Not at all* at −100 and *Extremely* at + 100. Pictures were presented in random order.

#### Balloon Analogue Risk Task

Our rationale for including the Balloon Analogue Risk Task (BART; Lejuez et al., 2002) was to reinforce our cover story. We informed participants that alcohol consumption would likely impair their performance on this task in order to increase their motivation to limit alcohol consumption during the taste test (see also Christiansen, Cole, & Field, 2012). Because this was not of primary interest, the data from this task were not analyzed. In the BART, participants click a button to pump up a balloon and collect a small reward (5 pence) for each pump. Rewards accrue with each balloon and can be banked at any time. If the participant chooses to bank the reward, that is the end of the trial, and a new balloon appears. However, if the participant opts to pump the balloon, the probability of it bursting increases. If the balloon bursts during a trial, all rewards accrued on that trial are lost, and the next trial (new balloon) begins. There were five trials in total. Participants were able to keep any rewards that they won.

### Procedure

Participants were invited to contact the researchers via phone or e-mail to check eligibility before attending. Upon arrival, participants attended a neutral laboratory (a conventional psychology testing laboratory with neutral décor, containing a desk, chairs, and a computer) and provided informed consent before completing a Two-Week Timeline Follow Back alcohol diary (TLFB; Sobell & Sobell, 1992), the Alcohol Use Disorders Identification Test (AUDIT; Babor, Higgins-Biddle, Saunders, & Monteiro, 2001); α = .70) to examine hazardous drinking, the Barratt Impulsivity Scale (BIS-11; Patton, Stanford, & Barratt, 1995; α = .65) to measure self-reported impulsivity, and finally the Temptation and Restraint Inventory (TRI; Collins & Lapp, 1992; αs ranged from .82 to .85) to measure motivation to reduce alcohol consumption. These measures were only completed during the first testing session. Following this, participants completed a baseline measure of craving (Approach and Avoidance of Alcohol Questionnaire [AAAQ]; McEvoy, Stritzke, French, Lang, & Ketterman, 2004; αs ranged from .84 to .89), the stimulus-value task, and the baseline stop-signal task, in that order. Participants were then randomized, using a random-number generator, to ICT or control groups before completing the relevant training task. Following completion of the task, they either remained in the neutral laboratory (no-context-shift condition) or were relocated to a seminaturalistic “bar lab” that resembled a British pub (context-shift condition) where they completed the posttraining assessment measures: the AAAQ, the stimulus-value task, and the stop-signal task (AAAQ analyses are presented in online supplemental materials). Participants then completed an ad libitum taste test in which they were provided with 300 ml of Heineken (5% alcohol by volume [ABV]), Budweiser (5% ABV), and Old Speckled Hen (6.5% ABV) in unmarked glasses (900 ml total) and instructed to rate each drink on a variety of gustatory dimensions while drinking as much or as little as they liked (see (Field & Jones, 2017). They were given 20 min to do this. Participants were also informed that following the ad libitum taste test, they would be completing a task where they could win small amounts of money and that alcohol could impair performance on this task. This was done to increase motivation to restrict alcohol consumption during the ad libitum taste test (Christiansen et al., 2012; Ostafin, Marlatt, & Greenwald, 2008). Following the ad libitum session, participants completed the BART. If this was their first session, their second session was then scheduled; the second session was identical to the first apart from the physical location of the posttraining assessments: If these were completed in the neutral laboratory (no-shift condition) in the first session, they were completed in the bar lab (context-shift condition) in the second session, and vice versa. At the end of their second session, participants completed a funneled debrief to examine their knowledge of the experiment with an open-ended question (“What was the purpose of the experiment?”) and two multiple-choice questions regarding the purpose of the training tasks and the taste test (see the online supplemental materials). Finally, participants were thanked, debriefed, and reimbursed £30 plus any money they won during the BART.

### Data Reduction and Analyses

We preprocessed reaction time (RT) data on go trials during the stop-signal task by removing probable anticipatory responses (any RTs <200 ms) and trials with errors. We also removed any RTs that were more than 3 standard deviations outside of the individual’s mean on each block (no-signal block, low-probability block, high-probability block) before computing the mean. This led to the removal of data from 8.98% of go trials.

For RT data on the training tasks, we also removed probable anticipatory responses (<200 ms) and incorrect responses (2.65%). We did not use a priori standard deviation cutoffs on RT data from the training task because we expected increased variability in RTs given that there were unequal trial numbers across groups (e.g., 10 alcohol go trials in ICT compared with 50 in control). Therefore, we report medians rather than means for summary data.^1^ We computed block-by-block summary data for each trial type.

A shorter stop-signal reaction time (SSRT) is indicative of better inhibitory control. To calculate the SSRT, we used the integration method with replacement of go omissions (failure to respond on go trials; note that in our preregistration, we stated that we would use the integration method, but we subsequently opted to follow the best practice of replacing go omissions based on a recently published consensus article for the stop-signal task [Verbruggen et al., 2019]). This method subtracts the mean stop-signal delay from the *n*th RT. First, we replaced go omissions with the slowest RT in the distribution. The *n*th RT was identified by ranking the go-trial RTs in the distribution (including incorrect responses) from fastest to slowest, then multiplying the number of go trials by the proportion of inhibitory failures. For example, if there were 90 go trials and participants failed to inhibit on 40% of stop trials, the *n*th RT was calculated as 90 × 0.4 = 36, and therefore the SSRT = the 36th go trial in the distribution. We did this separately for the low-probability and high-probability blocks. We removed any SSRTs that were negative (*N* = 6) and those where the average RTs on failed inhibition trials were slower than those on the go trials (*N* = 9), in line with guidance. Note that recently published simulations (which occurred after our data collection) suggest that more stop trials than were included in the low-signal block may be required to reliably estimate the stopping process (Verbruggen et al., 2019). Therefore, the SSRTs reported herein should be interpreted with caution.

We computed a measure of proactive slowing for the low-probability and high-probability blocks by subtracting the mean RT on go trials in the no-signal block from the mean go RT on those blocks. Slower RTs are therefore indicative of proactive slowing (Verbruggen & Logan, 2009). All relevant descriptive data from the stop-signal tasks (stop-signal delays, RTs for stop errors, etc.; see Verbruggen et al., 2019) are included in Table 2 of the online supplemental materials.

Where appropriate, we initially included a between-subjects factor of condition order (no context shift first vs. context shift first) for each ANOVA. If there were no main effects of order or interactions directly relevant to our hypotheses (interactions with Time × Condition), we reran the analysis without this factor to aid interpretation and increase statistical power. We used JASP (JASP Team, 2018) to calculate Bayes factors for our preregistered hypotheses based on uninformed priors. We report complete ANOVA tables for each hypothesis in Tables 3–6 of the online supplemental materials. Finally, test–retest reliability estimates were calculated using the intraclass correlation coefficient (ICC) using single measures from a two-way random model with absolute agreement.

## Results

### Participant Characteristics

Table 1 reports participant characteristics. On average, participants drank approximately 41.93 ± 24.64 units of alcohol in the 14 days prior to the first session of the experiment. This did not differ by experimental group, *t*(58) = 0.43, *p* = .67, *d* = 0.11, or gender (males = 42.15 ± 18.50; females = 41.83 ± 27.41), *t*(58) = 0.05, *p* = .96, *d* = 0.01. The average AUDIT score was 11.38 ± 4.69. AUDIT did not significantly differ across groups, *t*(58) = 1.13, *p* = .26, *d* = 0.29, or gender (males = 10.45 ± 3.91; females 11.85 ± 5.02), *t*(58) = 1.09, *p* = .28, *d* = 0.30.[Table-anchor tbl1]

### Performance on Training Tasks

Detailed analyses of RTs and accuracy for the training tasks are provided in the online supplemental materials. To summarize, there was no evidence of the formation of alcohol-inhibition associations over the course of training in either group, regardless of the training context. Inhibition accuracy was high (∼95%) during all training blocks (in line with similar ICT studies, as reviewed by Jones et al., 2016), and there was no evidence of an improvement in inhibition across successive training blocks in the ICT group. This may be attributed to the fact that each training block contained 200 trials, which is more than in previous ICT studies conducted in the laboratory (e.g., 80 trials in Houben et al., 2011, and Jones & Field, 2013). As such, optimal performance was reached early during the ICT/control tasks and maintained throughout.

### Hypothesis 1: ICT Will Reduce Alcohol Consumption in the Same Context But Also Following Context Shift Compared With Control (See Figure 1)

**Figure 1 fig1:**
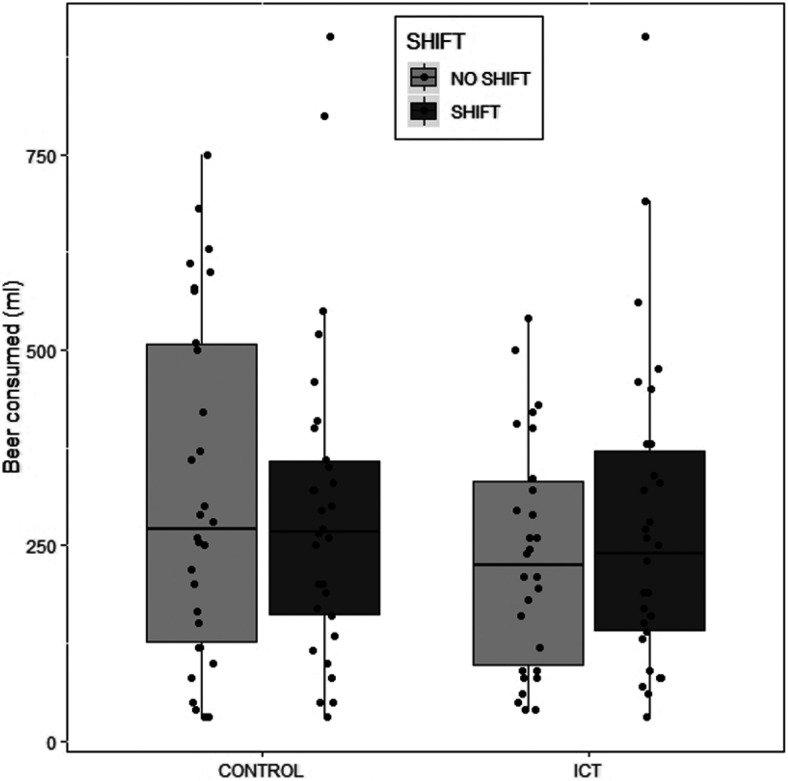
Amount of alcohol consumed, split by group and context shift.

The alcohol consumption data were not normally distributed (shift skewness statistic = 1.32 ± .31; no-shift skewness statistic = 0.77 ± .31). Therefore, we square-root transformed the data, which improved the distributions. However, because the interpretation of the data did not change, we present analyses on the nontransformed data here. Similarly, there were three outliers in the shift group who drank >800 ml in one session; the removal of these data points did not significantly alter the results presented here. Test–retest reliability was acceptable (ICC = .76).[Fig-anchor fig1]

The amount of alcohol consumed (in milliliters) at the end of each session for each participant was analyzed using a 2 (Group: ICT vs. Control) × 2 (Context: Shift vs. No Shift) mixed ANOVA. There were no main effects of group, *F*(1, 58) = 0.78, *p* = .38, η_p_^2^ = .01, or context, *F*(1, 58) < 0.01, *p* = .95, η_p_^2^ < .01. Furthermore, there was no significant Group × Context interaction, *F*(1, 58) = 1.81, *p* = .18, η_p_^2^ = .03. The Bayes factor for group was BF^10^ = 0.54, and the Group × Context interaction was BF^10^ = 0.06, indicating evidence in favor of the null hypothesis.

### Hypothesis 2: ICT Will Lead to Increased (a) Proactive Slowing and (b) Reactive Stopping Regardless of Context Compared With Control (See Table 2)

#### Proactive slowing

One participant had an outlying number of errors on go trials across blocks and time and so was removed from the analyses. Data were mostly normally distributed (skewness statistics ranged between .117 and .687, standard error [*SE*] = .314). Test–retest reliability was acceptable (ICC = .70). Proactive slowing was analyzed using a 2 (Group: ICT vs. Control) × 2 (Block: Low Probability vs. High Probability) × 2 (Context: Shift vs. No Shift) × 2 (Time: Baseline vs. Follow-Up) mixed ANOVA. The hypothesized Group × Time interaction was not significant, *F*(1, 56) = 0.88, *p* = .35, η_p_^2^ = .02, nor was the Group × Time × Context interaction, *F*(1, 56) = 1.40, *p* = .24, η_p_^2^ = .02. The Bayes factor for the Group × Time interaction was BF^10^ = 0.06, suggesting strong evidence for the null hypothesis. There was a main effect of block, *F*(1, 56) = 109.79, *p* < .001, η_p_^2^ = .66, demonstrating that proactive slowing was greater in the high-probability block (348.54 ms, *SE* = 24.38) compared with the low-probability block (254.73 ms, *SE* = 22.74). All other main effects or interactions that are relevant to our hypotheses were not statistically significant.[Table-anchor tbl2]

#### Reactive stopping

We examined SSRT using a 2 (Group: ICT vs. Control) × 2 (Context: Shift vs. No Shift) × 2 (Block: Low Probability vs. High Probability) × 2 (Time: Baseline vs. Follow-Up) mixed ANOVA. Test–retest reliability was poor (ICC = .20). The hypothesized Group × Time interaction, *F*(1, 44) = 0.05, *p* = .82, η_p_^2^ < .01, and the Group × Time × Context interaction were not significant, *F*(1, 44) = 1.23, *p* = .27, η_p_^2^ < .03.^2^ The Bayes factor for the Group × Time interaction was BF^10^ = 0.03, suggesting strong evidence for the null hypothesis. There were no other significant main effects or interactions directly relevant to our hypothesis.

### Hypothesis 3: ICT Will Increase the Devaluation of Alcohol-Related Stimuli in the Same Context But Also Following Context Shift Compared With Control

Data were missing on a case-wise basis from eight participants (six control; two ICT). Stimulus evaluations were not normally distributed (skewness statistics ranged from −0.68 to −0.90; *SE* = .32). Data transformations did not improve the distribution. Nonparametric tests did not alter the results; therefore, we report parametric tests here. Two participants had outlying values in at least two of the four measures of value; however, their removal did not significantly alter the results. Test–retest reliability was poor (ICC = .41).

Stimulus devaluation was analyzed using a 2 (Group: ICT vs. Control) × 2 (Context: Shift vs. No Shift) × 2 (Time: Baseline vs. Follow-Up) mixed ANOVA. Importantly, the hypothesized Group × Time interaction was not significant, *F*(1, 50) = 0.02, *p* = .88, η_p_^2^ < .01, nor was the Group × Time × Context interaction, *F*(1, 48) = 0.17, *p* = .68, η_p_^2^ < .01. The Bayes factor for the Group × Time interaction was BF^10^ = 0.17, which was supportive of the null hypothesis. There was a main effect of time, *F*(1, 50) = 4.14, *p* < .05, η_p_^2^ = .08, indicating that stimulus values increased at follow-up (19.27, *SE* = 4.48) compared with baseline (11.40, *SE* = 3.48). There was no main effect of group, *F*(1, 50) = 2.15, *p* = .15, η_p_^2^ = .04. There were no other significant main effects or interactions directly relevant to our hypothesis.

## Discussion

The aim of this study was to examine whether ICT that was intended to strengthen direct alcohol → inhibition associations in a neutral (lab-based) context would survive a context shift to a high-risk context (seminaturalistic bar). Our findings demonstrated no support for our hypotheses that ICT would reduce alcohol consumption, strengthen alcohol cue-inhibition associations, or lead to devaluation of alcohol-related stimuli, regardless of the testing context.

We hypothesized that ICT would lead to a reduction in alcohol consumption in the same context but also after a context shift. This hypothesis was not supported, and Bayes factors suggested support for the null. Furthermore, our data do not support previous studies demonstrating reductions in alcohol consumption following ICT when training and outcomes are measured in the same context (Bowley et al., 2013; Di Lemma & Field, 2017; Jones & Field, 2013).

We also failed to find support for our second hypothesis that ICT would lead to changes in both proactive and reactive inhibitory control processes to alcohol-related cues, and that these changes would survive a context shift. This is perhaps unsurprising because we have failed to demonstrate this previously (Jones, McGrath, et al., 2018), and there is limited evidence for the near or far transfer of inhibition training elsewhere (Enge et al., 2014; Talanow & Ettinger, 2018). This suggests that it is highly unlikely that any cognitive training procedures grounded in associative-learning principles would produce effects that persist across contexts under most practical circumstances (cf. cue exposure therapy of Conklin & Tiffany, 2002).

Finally, our findings did not support our final hypothesis that repeatedly inhibiting to alcohol cues would lead to stimulus devaluation. As such, there remains inconsistent evidence as to whether ICT influences stimulus evaluations (Veling et al., 2008), with the effects of food devaluation (Chen et al., 2016; Chen, Veling, Dijksterhuis, & Holland, 2018; Lawrence et al., 2015) seemingly being more robust than those of alcohol devaluation (Houben et al., 2012).

We note the following limitations of our study. We were powered to detect a medium effect size for a Context × Group interaction effect on ad libitum alcohol consumption (*d* = .50; slightly larger than current estimates of pooled estimates from a recent meta-analysis of *d* = .43 for the main effect of ICT on alcohol consumption in laboratory settings^3^ [Jones, Di Lemma, et al., 2016]). However, we were only able to reliably detect an effect size of *d* = .65 for the (between-subjects) main effect of group (at 80% power). Furthermore, the effect sizes on inhibitory control and stimulus devaluation are less clear and likely to be considerably smaller (e.g., *d*s ranging from .16 to .37 in Chen et al., 2018; BF^01^ = 0.23, supporting the null hypothesis, in Adams et al., 2017), and our analyses required more complex three- and four-way interactions because they incorporated the effects of time and proactive control. Nevertheless, our Bayes factors were broadly supportive of the null hypothesis, suggesting our data were sensitive enough to support our inferences. Second, we did not administer ICT in a high-risk drinking environment (e.g., bar or pub). ICT may still have therapeutic benefits if administered in environments in which alcohol is present, and future studies may consider utilizing ecological momentary intervention techniques to administer ICT (Blackburne, Rodriguez, & Johnstone, 2016). Third, it is possible that our measure of proactive slowing reflects increased attention to alcohol-related cues, which act as a signal for inhibition (in the ICT group) rather than strategic slowing. We note that previous ICT studies have demonstrated decreases (rather than increases) in selective attention to trained cues (Stice, Lawrence, Kemps, & Veling, 2016). However, future studies should attempt to disentangle proactive slowing from increased attention directly. Finally, we did not measure ad libitum alcohol consumption at baseline (before ICT), which complicates the interpretation of the absence of the hypothesized group difference in alcohol consumption after training.

Given the discrepancies with previous findings, future ICT studies should also directly compare the behavioral effects of simplistic ICT training paradigms with the more sophisticated paradigm that was used in the present study. However, methodological issues aside, it is important to interpret the present findings in the context of the broader literature on ICT and related cognitive-bias-modification interventions, which have weak and inconsistent effects on substance use (Boffo et al., 2019; Cristea, Kok, & Cuijpers, 2016).

How, then, should we interpret the failure to support any of our hypotheses, in order to best inform the field moving forward? If changes in inhibitory control to alcohol-related cues (alcohol → inhibition associations) are a candidate mechanism of ICT, then one potential explanation for our failure to replicate previously published effects is the absence of any measurable change in inhibitory control to alcohol-related cues during or immediately after training. It is clear that our ICT design did not effectively train alcohol → inhibition associations, and there are multiple potential explanations for this. First, we used a control group of 50% alcohol inhibition contingencies, rather than reversed contingencies (10% alcohol inhibition; cf. Di Lemma & Field, 2017; Jones & Field, 2013). Reversed-contingency designs are useful in proof-of-concept designs to identify/amplify a target mechanism. However, they are likely to be uninformative (and unethical) comparison conditions in subsequent randomized controlled trials because they may increase approach behaviors to alcohol (see Bakkour et al., 2016; Schonberg et al., 2014). As such, proof-of-concept studies using reversed-contingency designs could inadvertently generate inflated estimates of the behavioral effects of ICT. Second, it is possible that our training paradigm, although designed to amplify direct alcohol → inhibition associations, was too complex because participants had to repeatedly learn different task rules. This might have a counterproductive effect, particularly in heavy alcohol consumers (and individuals with alcohol use disorder), who demonstrate cognitive impairments and inability to concentrate (Bernardin, Maheut-Bosser, & Paille, 2014; Tembo, Burns, & Kalembo, 2017). Third, the reliability of the inhibition errors during the training task and SSRTs was suboptimal (see also Wostmann et al., 2013). The poor reliability of cognitive tasks inevitably reduces confidence in any inferences based on group differences in those tasks (Rodebaugh et al., 2016). Future research should conduct rigorous preliminary work to ensure that ICT training paradigms robustly promote the learning of cue-inhibition associations before progressing to investigate the behavioral effects of such training.

Assuming that a change in alcohol-related inhibitory control (alcohol → inhibition associations) is the proposed mechanism through which ICT causes reductions in alcohol consumption, we cannot conclude that ICT is an ineffective tool for the reduction of alcohol consumption based on the present findings. Instead, we must conclude that our training was ineffective at changing the target construct (cf. discussions by Boffo et al., 2019; Grafton et al., 2017; Sheeran, Klein, & Rothman, 2017). However, it is worth noting that many ICT studies have failed to test or report the changes in alcohol → inhibition associations following training (Bowley et al., 2013; Houben et al., 2011) or found no changes in alcohol → inhibition associations but have nonetheless detected reductions in alcohol consumption (Di Lemma & Field, 2017; Houben et al., 2012). This further complicates the broader interpretation of ICT effects. It is also possible that ICT training conducted in a single brief laboratory session is not sufficient to promote associative learning, particularly when the task complexity is increased, as it was in the present study.

An alternative viewpoint suggests that the change in candidate mechanisms of action is irrelevant when testing interventions (particularly using gold-standard intention-to-treat principles), and if we do not observe a robust reduction in drinking behavior, then we should interpret ICT as a failed intervention with limited clinical utility in this population (Cristea, 2018; Cristea, Kok, & Cuijpers, 2017). If we follow this line of reasoning, then repeatedly testing failed interventions for unknown mechanisms or boundary conditions serves only to increase wasteful research chasing small, unstable effects.

To conclude, with this preregistered study, we add to the growing body of evidence that ICT administered in the laboratory may not yield robust reductions in alcohol consumption in heavy drinkers. Although ICT has proved a popular area of study, the recent emergence of negative results means that future researchers may wish to abandon ICT in favor of alternative interventions that may translate outside of the laboratory environment.

## Supplementary Material

10.1037/adb0000580.supp

## Figures and Tables

**Table 1 tbl1:** Demographic Characteristics of the Sample, Split by Experimental Group

Characteristic	Control (*N* = 30)	ICT (*N* = 30)
Age	25.70 (7.55)	24.97 (6.11)
Gender (F:M)	9: 21	11: 19
AUDIT	12.07 (5.13)	10.70 (4.19)
Units cons.	43.30 (28.18)	40.57 (20.91)
TRI CBC	17.7 (8.89)	16.47 (9.53)
TRI CEP	29.90 (12.42)	24.93 (12.12)
BIS total	70.77 (8.80)	67.97 (9.74)
*Note*. ICT = inhibitory control training; AUDIT = Alcohol Use Disorders Identification Test; Units cons. = alcohol consumed in previous fortnight; TRI CBC = Temptation and Restraint Inventory, Cognitive Behavioral Control Subscale; TRI CEP = Temptation and Restraint Inventory, Cognitive Emotional Preoccupation Subscale; BIS = Barratt Impulsivity Scale. Continuous variables are means and standard deviations.		

**Table 2 tbl2:** Dependent Variables (Inhibitory Control Processes and Stimulus Value) Split by Group, Time, and Context

Variable	Control	ICT
Baseline no shift		
Proactive slowing (high)	345.88 (213.78)	389.02 (197.88)
Proactive slowing (low)	234.03 (193.23)	239.21 (183.24)
SSRT (high)	200.09 (74.73)	217.78 (78.62)
SSRT (low)	226.25 (83.45)	216.07 (67.72)
Value	17.88 (30.11)	9.57 (38.27)
Baseline shift		
Proactive slowing (high)	331.79 (210.64)	394.74 (185.28)
Proactive slowing (low)	258.11 (209.26)	323.19 (191.01)
SSRT (high)	226.90 (57.39)	231.78 (97.03)
SSRT (low)	224.44 (70.34)	231.18 (50.31)
Value	3.70 (38.21)	6.58 (34.50)
Follow-up no shift		
Proactive slowing (high)	289.31 (188.42)	417.57 (218.44)
Proactive slowing (low)	214.53 (209.69)	321.25 (204.09)
SSRT (high)	218.59 (74.34)	220.30 (68.89)
SSRT (low)	235.18 (80.51)	230.84 (81.50)
Value	17.38 (38.84)	19.94 (36.99)
Follow-up shift		
Proactive slowing (high)	316.82 (234.16)	328.87 (200.04)
Proactive slowing (low)	241.59 (208.09)	242.01 (218.09)
SSRT (high)	234.55 (69.41)	258.83 (67.72)
SSRT (low)	235.54 (69.20)	272.68 (71.33)
Value	17.21 (37.51)	13.71 (36.29)
*Note*. ICT = inhibitory control training; SSRT = stop-signal reaction time. Values are means and standard deviations.		

## Footnotes

1 Note that we did not preregister an analysis strategy for the training data.

2 Note that the reduction in denominator degrees of freedom in this model is due to a number of negative SSRTs removed from our analyses.

3 At the time of preregistration, we were unaware of any studies that had examined a change in ad lib consumption over time, thus providing an accurate between–within interaction effect size.
