# Supplementary material for: Does Alcohol Cue Inhibitory Control Training Survive a Context Shift?
Source: Psychol Addict Behav. 2020 Apr 13;34(7):783–92. doi: 10.1037/adb0000580 (PMC7650386; doi:10.1037/adb0000580)
Supplement: Supplementary file 1 [file adb0000580Supplemental.docx]

**Online Supplementary Materials for ‘Does alcohol cue Inhibitory Control Training survive a context shift?**

**Contents:**

1. **Performance on the training tasks**
2. **Changes in craving following ICT vs control**
3. **Examination of responses to funnelled debrief questionnaire**
4. **Covariate analyses examining influences (previous alcohol use, hazardous drinking and motivation) on ad-libitum consumption following ICT vs Control**
5. **Supplementary tables for craving scores and ANOVAS for pre-registered analyses.**
6. **Performance on the training tasks**

*Reaction times*: Median reaction time to different cues (alcohol / neutral) across training blocks demonstrated reasonable reliability (α’s ranged from .74 - .94). For median Go RTs we conducted a 2 (image: Alcohol vs Neutral) x 2 (Block: Block 1 vs 4) x 2 (Context: Shift vs No Shift) x 2 (group: ICT vs Control) mixed ANOVA. The hypothesised image * group * block (*F*(1,58) = 2.34, *p* =.13, η_p_^2^ = .04), and image * group * block * context interactions (*F*(1,58) = 1.52, *p* =.22, η_p_^2^ = .03) were not significant, suggesting reaction times to alcohol/neutral cues did not change over the course of training blocks or contexts.

There was a significant main effect of image (*F*(1,58) = 65.23, *p* < .01, η_p_^2^ = .53), subsumed under an image * group interaction (*F*(1,58) =6.18, *p* = .02, η_p_^2^ = .10). ***Reaction times to alcohol related cues were significantly slower than reaction times to neutral cues in both the ICT group (Alcohol = 589.91 ms, SE = 11.17; Neutral = 552.47, SE = 9.11; t(29) = 5.63, p < .01, d = 1.07) and the control group (Alcohol = 577.38 ms, SE = 9.81; Neutral = 557.56, SE = 10.30; t(29) = 8.13, p < .01, d = 1.49)****.* ***Contrary to expectations the effect size for the difference was larger in the control group compared to the ICT group, however the median differences (alcohol RT – control RT) were larger in the ICT group (37.44 ms) compared to control (19.82 ms). The reason for the larger effect sizes, but smaller mean difference is due to increased variability of reaction times (specifically for alcohol-related cues) in the ICT group.’***  There were no significant differences between alcohol (*t*(58) = 0.84, *p* = .40, *d* = 0.22) and neutral images (*t*(58) = 0.37, *p* = .71, *d* = 0.10) across groups^[[1]](#footnote-1)^. There was a main effect of order (*F*(1, 56) = 4.81, *p* = .03, η_p_^2^ = .08), indicating faster reaction times in individuals who did the ‘no context shift’ condition first (553.73 ms, SE = 10.05; 583.45, SE = 583.45). However, there were no relevant interactions with group, or time (*Fs* < 2.83, *ps* > .09) so we did not analyse this further. There was also a significant main effect of Block (*F*(1, 58) = 9.13, *p* < .01, η_p_^2^ = .14), demonstrating that reaction times were faster on block 4 (561.75 ms, SE = 7.42) compared to block 1 (576.91, SE = 7.31; *p* = .003). The main effects of group (*F*(1,58) = 0.07, *p* = .79, η_p_^2^ = .001), and context (*F*(1,58) = 1.06, p = .31, η_p_^2^ = .02) were not significant. There were no other significant main effects or interactions (*Fs* < 2.43, *ps* > .124).

As we did not pre-register an analysis plan for training effects and there is limited evidence by which to establish a plausible effect size of ICT on inhibitory control processes, it is possible we were underpowered to identify complex 3 and 4-way interactions. Therefore, we examined the simple effect of ICT on alcohol-related reaction times in the no context shift condition only, to clarify whether training improved inhibitory control to alcohol in line with previous research (Jones & Field, 2012). The paired samples t-test comparing median reaction times from blocks 1 and 4 in the ICT group demonstrated no significant slowing of reaction times to alcohol-related cues over the course of training (block 1 = 593.13 ms ± 77.70, block 4 = 587.14 ms ± 79.15: *t* (29) = 0.43, *p* = .67, *d* = .08), suggesting ICT did not significantly slow reaction times to alcohol-related cues.

*Inhibition accuracy:* Reliability for inhibition accuracy was poor across blocks and cues (α’s ranging from .26 - .71). For the proportion of correct inhibition we conducted a 2 (image: Alcohol vs Neutral) x 2 (Block: Block 1 vs 4) x 2 (Context: Shift vs No Shift) x 2 (group: ICT vs Control) mixed ANOVA. The hypothesised image * group * block (*F*(1,58) = 0.02 ,*p* =.88, η_p_^2^ < .01), and image * group * block * context interaction interactions (*F*(1,58) = 2.37, *p* =.13, η_p_^2^ = .04) were not significant, suggesting inhibition accuracy to alcohol/neutral cues did not change over the course of training blocks or contexts.

There was a main effect of image (*F*(1,58) = 10.63, *p* < .01, η_p_^2^ = .16) and a main effect of group (*F*(1, 58) = 4.83, *p* = .03, η_p_^2^= .08), which were subsumed under a significant image * group interaction (*F*(1,58) = 34.07, *p* < . 01, η_p_^2^ = .37). In the ICT group, inhibition success was significantly greater to alcohol (96.13%, SE = 0.57) compared to neutral cues (90.83%, SE = 1.24; *t*(29) = 4.92, *p* < . 01, *d* = .90). In the control group, inhibition to neutral cues (96.33%, SE = .59) was significantly better than inhibition to alcohol cues (94.83%, SE = 0.56; *t*(29) = 3.38, *p* < .01, *d* = 0.62 ). The between-group difference was statistically significant for neutral cues (*t*(58) = 4.02, *p* < .001, *d* = 1.04), but not for alcohol cues (*t*(58) = 1.63, *p* = .11, *d* = 0.42). There were no other significant main effects or interaction (*Fs* < 3.37, *ps* > .07). The main effects of block (*F*(1, 58) = 2.78, *p* = .100, η_p_^2^ = .05) and context (*F*(1, 58) = 3.27, *p* = .08, η_p_^2^ = .05) were not significant.

As with the reaction time data we examined the simple within-subjects contrast of the training effect on inhibition errors to alcohol related cues in the ICT group in the no context condition only. The paired samples t-test comparing inhibition success in block 1 (97.19% ± 3.07) with those in block 4 (95.59% ± 4.03; t (29) = 1.87, p = .07, d = -.34) was not significant, and the pattern of results was in the opposite direction than expected. Taken together, the findings from reaction times and inhibition errors suggest that our ICT training was ineffective at establishing alcohol-inhibition associations.

1. **Changes in craving (see Supplementary table 1)**

Changes in craving due to ICT were analysed using a 3 (scale: Inclined, Obsessed, Resolved) x 2 (group: ICT vs Control) x2 (context: Shift vs No Shift) x 2 (time: Baseline vs Follow-up) mixed ANOVA. There was a main effect of scale (F(2, 51) = 113.99, p < .001, np2 = .817), indicating scores on the inclined subscale were greater than the obsessed (p < .001) and resolved subscales (p < .001). Scores on the obsessed subscale were not significantly different from the avoidant subscale (p = .174). There was also a significant scale * time interaction (F(2, 104) = 6.25, p = .003, np2 = .107) and a scale * context * GROUP interaction (F(2,104) = 3.96, p = .022). However, as we did not observe a significant interaction with time and group (scale * group * time interaction (F(2, 104) = 0.35, p = .709, np2 = .007); scale * time * group * context interaction (F(2, 104) = 2.17, p = .119, np2 = .040) we did not examine these interactions further.

1. **Funnelled debrief.**

***Participants completed a funnelled debrief which consisted of an open-ended question ‘What was the purpose of the experiment?’, and two multiple choice questions ‘What was the purpose of the computerised tasks’ and ‘What was the purpose of the taste test?’.***

***No participants completely guessed the nature of the experiment in the open-ended question (some example responses were ‘To investigate how alcohol affects my behaviour and whether seeing images of alcohol affects my accuracy on a task’ and ‘Perhaps seeing it being in a pub environment made cravings for alcohol stronger in comparison to another context.’). There were 5 possible answers for each multiple-choice question and participants were allowed to tick more than one response (see Jones et al, 2011) for similar designs).***

***The correct multiple-choice answer for ‘What was the purpose of the computerised task’ was ‘To train me to inhibit to alcohol / press to neutral cues’ in the ICT group. There was no directly comparable correct answer for the control group. Nine participants in the ICT group correctly guessed the response.***

***The correct multiple choice answer for ‘What was the purpose of the taste test?’ was ‘To Measure how much alcohol I drank in response to the computer task’. Twenty-four participants correctly guessed this response, with no significant difference between groups (14 in the ICT group, 10 in the Control group: X2(1) = 1.11, p = .292).***

1. ***Potential influencers of ICT effect on ad-libitum alcohol consumption***

***We performed exploratory analyses to examine whether alcohol use, severity or motivation to reduce consumption might have influenced the results of our primary analysis.***

***Alcohol use***

***We re-ran our main analysis (2: Context x 2: Group mixed ANOVA) on ad-libitum alcohol consumption, with the inclusion of previous alcohol use as a covariate. The hypothesised main effect of condition (F(1, 56) = 0.47, p = .498, np2 = .008), the context * condition interaction (F(1, 56) = 0.04, p = .846, np2 = .001), the condition * covariate (F(1, 56) = 0.09, p = .763, np2 = .002) and the context * condition * covariate interactions (F(1,56) = 0.26, p = .609, np2 = .005) were not significant.***

***Drinking severity (AUDIT)***

***We re-ran our main analysis (2: Context x 2: Group mixed ANOVA) on ad-libitum alcohol consumption, with the inclusion of AUDIT scores as a covariate. The hypothesised main effect of condition (F(1, 56) = 0.03, p = .868, np2 < .001), the context * condition interaction (F(1, 56) = 0.41, p = .527, np2 = .007), the condition * covariate (F(1, 56) = 0.03, p = .872, np2 < .001) and the context * condition * covariate interactions (F(1,56) = 0.06, p = .813, np2 = .001) were not significant.***

***Motivation (TRI – Cognitive Behavioural Control subscale)***

***We re-ran our main analysis (2: Context x 2: Group mixed ANOVA) on ad-libitum alcohol consumption, with the inclusion of TRI-CBC scores as a covariate. The hypothesised main effect of condition (F(1, 56) = 0.01, p = .971, np2 < .001), the condition * covariate (F(1, 56) = 0.16, p = .694, np2 = .003) and the context * condition * covariate interactions (F(1,56) = 2.83, p = .098, np2 = .048) were not significant. The context * condition interaction was marginally significant (F(1, 56) = 4.37, p = .041, np2 = .072). The effect condition was not significant in either context (no shift (t(58) = 1.30, p = .200; shift (t(58) = 0.36, p = .718).***

**Supplementary table 1: AAAQ craving subscales split by GROUP, time and context. Values are means (± standard deviations).**

**Baseline Follow up**

***Control ICT Control ICT***

*Same context*

Inclined 4.55 ± 1.72 4.14 ± 1.72 4.54 ± 2.03 4.59 ± 2.12

Obsessed 1.33 ± 1.33 1.14 ± 1.50 1.62 ± 2.08 1.56 ± 1.90

Resolved 1.03 ± 1.13 1.43 ± 1.58 0.96 ± 1.23 1.13 ± 1.33

*Shift in context*

Inclined 4.97 ± 1.77 4.13 ± 2.08 5.27 ± 1.78 4.21 ± 2.16

Obsessed 1.47 ± 1.71 1.09 ± 1.46 1.80 ± 1.93 1.33 ± 1.78

Resolved 1.01 ± 1.18 1.35 ± 1.61 0.98 ± 1.08 1.21 ± 1.60

*Legend: Inclined = AAAQ inclined-indulgent subscale; Obsessed =AAAQ obsessed-compelled subscale; Resolved = AAAQ resolved-regulated subscale.*

**Supplementary table 2. Descriptive information for the Stop Signal tasks. Values are Means (± standard deviations)**

**Control ICT**

**Baseline (shift) High Low High Low**

**Go incorrect** 10.56 (12.78) 7.97 (15.38) 9.10 (8.48) 4.03 (4.90**)**

**p(respond|signal)** 42.83 (9.59) 46.67 (10.21) 45.28 (4.08) 48.44 (12.92)

**SSD** 740.26 (202.99) 683.11 (193.25) 658.14 (204.65) 666.86 (161.41)

**RT on failed stop** 740.26 (202.99) 683.11 (193.25) 783.98 (185.20) 666.86 (161.41)

**Go RTs** 878.63 (217.14) 787.84 (215.91) 910.19 (181.38) 760.38 (164.69)

**Go RTs No signal** 523.87 (116.73) 521.17 (109.21)

**Baseline (no shift)**

**Go incorrect** 9.67 (8.82) 7.80 (12.50) 11.8 (11.56) 10.20 (17.50)

**p(respond|signal)** 44.56 (6.13) 45.00 (10.68) 42.06 (8.71) 46.33 (8.94)

**SSD** 678.39 (223.16) 732.88 (189.40) 704.03 (192.42) 652.72 (241.80)

**RT on failed stop** 815.25 (163.31) 765.37 (173.51) 815.25 (163.31) 765.37 (173.51)

**Go RTs** 945.16 (178.25) 873.61 (193.59) 945.16 (178.25) 873.61 (193.59)

**Go RTs No Signal** 550.42 (136.99) 550.42 (136.99)

**Follow up (shift)**

**Go incorrect** 8.77 (12.52) 7.27 (15.27) 6.07 (5.96) 4.13 (4.16)

**p(respond|signal)** 45.28 (10.07) 45.67 (11.25) 45.72 (4.19) 48.33 (6.87)

**SSD** 601.33 (277.28) 522.06 (252.14) 596.39 (193.48) 501.72 (200.48)

**RT on failed stop** 708.96 (243.60) 654.67 (204.94) 751.15 (179.30) 682.59 (163.57)

**Go RTs** 855.24 (236.65) 782.85 (217.79) 871.28 (178.75) 784.41 (181.74)

**Go RTs No Signal** 525.09 (123.34) 542.40 (139.23)

**Follow up (no shift)**

**Go incorrect** 7.83 (9.92) 8.93 (16.31) 9.83 (9.04) 7.30 (13.50)

**p(respond|signal)** 43.88 (7.09) 48.89 (8.81) 44.22 (7.13) 44.78 (10.99)

**SSD** 581.64 (226.17) 540.61 (281.13) 684.50 (237.55) 575.56 (229.33)

**RT on failed stop**  712.01 (184.36) 643.22 (177.69) 799.69 (213.13) 712.98 (198.64)

**Go RTs** 833.15 (200.86) 762.96 (219.86) 925.45 (200.07) 829.13 (204.16)

**Go RTs No Signal** 530.75 (126.86) 507.88 (115.12)

*Legend: p(respond|signal) = probability of responding to a Stop Signal (inhibition error); RTs = Reaction times; SSD = Stop signal delay*

**Supplementary Table 3: ANOVA table for Hypothesis 1 (ad-libitum consumption)**

**Sum of Squares Mean Squares F - Value P- value η_p_^2^**

**CONTEXT**  35.21 35.21 <0.01 .952 .000

**CONTEXT * GROUP**  17160.21 17160.21 1.81 .184 .030

**GROUP**  54826.88 54826.88 0.78 .382 .013

**Supplementary Table 4: ANOVA table for Hypothesis 2a (proactive control)**

**Sum of Squares Mean Squares F - Value P- value η_p_^2^**

**BLOCK**  1019663.61 1019663.61 109.79 <.000 .662

**BLOCK * GROUP**  6213.80 6213.78 0.67 .417 .012

**CONTEXT**  62962.85 62962.85 3.06 .086 .052

**CONTEXT * GROUP**  195708.93 195708.93 9.51 .003 .145

**TIME**  46378.84 46378.84 2.98 .090 .051

**TIME * GROUP**  13750.65 13750.65 0.88 .351 .016

**BLOCK * CONTEXT**  15897.49 15897.49 2.84 .098 .048

**BLOCK * CONTEXT * GROUP** 3488.01 3488.01 0.62 .433 .011

**BLOCK * TIME**  7376.07 7376.07 1.36 .248 .024

**BLOCK * TIME * GROUP**  284.67 284.67 0.053 .820 .001

**CONTEXT * TIME**  1948.85 1948.85 0.10 .755 .002

**CONTEXT * TIME * GROUP** 27676.34 27676.34 1.40 .242 .024

**BLOCK * CONTEXT * TIME** 34762.40 34762.40 8.52 .005 .132

**BLOCK * CONTEXT * TIME**

*** GROUP**  2458.07 2458.07 0.60 .441 .011

**GROUP**  426850.79 426850.75 1.72 .195 .030

**Supplementary Table 5: ANOVA table for Hypothesis 2b (reactive control: SSRTs)**

**Sum of Squares Mean Squares F - Value P- value η_p_^2^**

**CONTEXT**  42118.82 42118.82 7.11 .011 .136

**CONTEXT * GROUP**  18023.29 18023.29 3.04 .088 .063

**BLOCK**  5132.66 5132.66 1.57 .217 .034

**BLOCK * GROUP**  756.34 756.34 0.23 .633 .005

**TIME**  34500.68 34500.68 9.34 .004 .172

**TIME * GROUP**  187.41 187.41 0.05 .823 .001

**CONTEXT * BLOCK**  465.42 465.42 0.09 .769 .002

**CONTEXT * BLOCK * GROUP** 5737.20 5737.20 1.08 .304 .023

**CONTEXT * TIME**  1758.20 1758.20 0.44 .511 .010

**CONTEXT * TIME * GROUP** 4908.71 4908.71 1.23 .274 .027

**BLOCK * TIME**  1549.66 1549.66 0.27 .607 .006

**BLOCK * TIME * GROUP**  511.73 511.73 0.09 .768 .002

**CONTEXT * BLOCK * TIME** 977.89 977.89 0.28 .600 .006

**CONTEXT * BLOCK * TIME ***

**GROUP** 1504.94 1504.94 0.43 .516 .009

**GROUP**  3751.46 3751.46 0.30 .590 .007

**Supplementary Table 6: ANOVA table for Hypothesis 3 (stimulus values)**

**Sum of Squares Mean Squares F - Value P- value η_p_^2^**

**CONTEXT**  1097.09 1097.09 1.38 .246 .027

**CONTEXT * GROUP** 65.49 65.49 0.08 .775 .002

**TIME**  3206.21 3206.21 4.14 .047 .076

**TIME * GROUP**  16.70 16.70 0.02 .884 .000

**CONTEXT * TIME**  61.40 61.40 0.12 .735 .002

**CONTEXT * TIME * GROUP** 92.12 92.18 0.17 .678 .003

**GROUP**  5494.69 2553.95 2.15 .149 .041

*Legend for supplementary tables 3- 6:Block= High Signal or Low Signal; Context = Shift or No Shift in Context.; Group = ICT vs Control; Time = Baseline or Follow-up (after ICT/Control).*

1. Note that we would only be reliably powered to detect medium-to-large main effects of training group (see limitations in main manuscript). [↑](#footnote-ref-1)
